# Screening of potent neutralizing antibodies against SARS-CoV-2 using convalescent patients-derived phage-display libraries

**DOI:** 10.1038/s41421-021-00295-w

**Published:** 2021-07-27

**Authors:** Yongbing Pan, Jianhui Du, Jia Liu, Hai Wu, Fang Gui, Nan Zhang, Xiaojie Deng, Gang Song, Yufeng Li, Jia Lu, Xiaoli Wu, ShanShan Zhan, Zhaofei Jing, Jiong Wang, Yimin Yang, Jianbang Liu, Ying Chen, Qin Chen, Huanyu Zhang, Hengrui Hu, Kai Duan, Manli Wang, Qisheng Wang, Xiaoming Yang

**Affiliations:** 1 National Engineering Technology Research Center for Combined Vaccines, Wuhan Institute of Biological Products Co. Ltd., Wuhan, Hubei China; 2 State Key Laboratory of Virology, Wuhan Institute of Virology, Center for Biosafety Mega-Science, Chinese Academy of Sciences, Wuhan, Hubei China; 3 Shanghai Institute of Applied Physics, Chinese Academy of Sciences, Shanghai, China; 4 University of Chinese Academy of Sciences, Beijing, China; 5 Shanghai Synchrotron Radiation Facility, Shanghai Advanced Research Institute, Chinese Academy of Sciences, Shanghai, China; 6 China National Biotec Group Company Limited, Beijing, China

**Keywords:** Immunology, Structural biology

## Abstract

As the severe acute respiratory syndrome coronavirus 2 (SARS-CoV-2) continues to threaten public health worldwide, the development of effective interventions is urgently needed. Neutralizing antibodies (nAbs) have great potential for the prevention and treatment of SARS-CoV-2 infection. In this study, ten nAbs were isolated from two phage-display immune libraries constructed from the pooled PBMCs of eight COVID-19 convalescent patients. Eight of them, consisting of heavy chains encoded by the immunoglobulin heavy-chain gene-variable region (*IGHV*)3-66 or *IGHV*3-53 genes, recognized the same epitope on the receptor-binding domain (RBD), while the remaining two bound to different epitopes. Among the ten antibodies, 2B11 exhibited the highest affinity and neutralization potency against the original wild-type (WT) SARS-CoV-2 virus (K_D_ = 4.76 nM for the S1 protein, IC_50_ = 6 ng/mL for pseudoviruses, and IC_50_ = 1 ng/mL for authentic viruses), and potent neutralizing ability against B.1.1.7 pseudoviruses. Furthermore, 1E10, targeting a distinct epitope on RBD, exhibited different neutralization efficiency against WT SARS-CoV-2 and its variants B.1.1.7, B.1.351, and P.1. The crystal structure of the 2B11–RBD complexes revealed that the epitope of 2B11 highly overlaps with the ACE2-binding site. The in vivo experiment of 2B11 using AdV5-hACE2-transduced mice showed encouraging therapeutic and prophylactic efficacy against SARS-CoV-2. Taken together, our results suggest that the highly potent SARS-CoV-2-neutralizing antibody, 2B11, could be used against the WT SARS-CoV-2 and B.1.1.7 variant, or in combination with a different epitope-targeted neutralizing antibody, such as 1E10, against SARS-CoV-2 variants.

## Introduction

The coronavirus disease 2019 (COVID-19) pandemic caused by the severe acute respiratory syndrome coronavirus 2 (SARS-CoV-2) poses a severe threat to public health. The high-transmission capacity and potential severe outcomes upon infection highlight the need to develop effective preventive and therapeutic approaches to contain the pandemic. The structure of the SARS-CoV-2 spike (S) protein and its interaction with angiotensin-converting enzyme 2 (ACE2) on the surface of host cells have been well studied;^1^ the homotrimeric S glycoprotein undergoes significant conformational changes, from pre- to post fusion, referred to as the “down” and “up” states, upon recognition/binding to ACE2^1^. The SARS-CoV-2 S protein consists of two functional subunits, S1, which binds to the target receptor in the host cells, and C-terminal S2 (separated by the Furin and S2′ cleavage sites recognized by host proteases), important for the fusion of the viral and cellular membranes^2,3^. Importantly, blockage of the receptor-binding domain (RBD) of S1 with neutralizing antibodies (nAbs), resulted in inhibiting the entry of SARS-CoV-2 into host cells. Of note, the S trimer contains 66 N-linked and three O-linked glycan sequons^4–6^, which might play a critical role in immune escape via the masking of immunogenic protein epitopes. In addition, the emergence of SARS-CoV-2 variants also contributes to immune escape events; in fact, the newly emerged SARS-CoV-2 variants B.1.1.7 (also known as 501Y.V1) in the UK, B.1.351 (also known as 501Y.V2) in South Africa, P.1 (also known as 501Y.V3) in Brazil, with different amino acid mutations, including D614G, N501Y, E484K, and K417N, were associated with immune escape and enhanced transmission/increased infectivity^7^.

Convalescent plasma with high concentrations of virus-specific immunoglobulins or recombinant fully human antibodies were shown to decrease the viral loads and reduce the mortality in patients infected with the Middle East Respiratory Syndrome Coronavirus (MERS-CoV)^8^, human immunodeficiency virus (HIV)^9^, Ebola virus (EBOV)^10^, and most recently SARS-CoV-2^11^, confirming the efficacy of nAbs as prophylactic and therapeutic agents against viral infection. To date, most of the SARS-CoV-2 nAbs identified were derived from peripheral blood mononuclear cells (PBMC)s of convalescent COVID-19 patients^12,13^, or transgenic mice^14^ in the context of single B-cell sorting^12,14^. The phage-display technology, an efficient and robust platform for antibody discovery, has been widely used in the generation of many recombinant antibodies or fragments for both the diagnosis and treatment of infections caused by several viruses, including influenza virus^15^, EBOV^16^, HIV^17^, Herpes simplex virus^18^, rabies virus^19^, hepatitis B virus^20^, West Nile virus^21^, and SARS-CoV^22^. Although there are several reports^23–25^ on the screening of anti-SARS-CoV-2 nAbs using phage-display platforms, most of them were constructed from naive humans or immunized animals; the potential of convalescent patient-derived immune phage-display libraries for the generation of nAbs remains to be explored.

In this study, using two phage-display immune libraries based on pooled PBMCs from eight convalescent patients, we obtained ten SARS-CoV-2 nAbs. Among them, 2B11 exhibited the highest potent neutralizing ability against the original wild-type (WT) SARS-CoV-2 virus and its variants B.1.1.7. Moreover, 2B11 showed encouraging therapeutic and prophylactic efficacy in adenovirus 5 expressing human ACE2 (AdV5-hACE2) transduced mice infected with WT SARS-CoV-2. In addition, another nAb targeting a distinct epitope on RBD, 1E10, exhibited a complete inhibition potential against the emergent SARS-CoV-2 variants B.1.1.7, B.1.351, and P.1 in vitro. Overall, using a convalescent patients-derived phage-display approach, we obtained the potent nAb 2B11 and the broad-spectrum nAb 1E10 as potential to be combination therapy against WT SARS-CoV-2 and its variants.

## Results

### Collection of blood samples from convalescent patients and determination of the anti-RBD titers

**Fig. 1:**
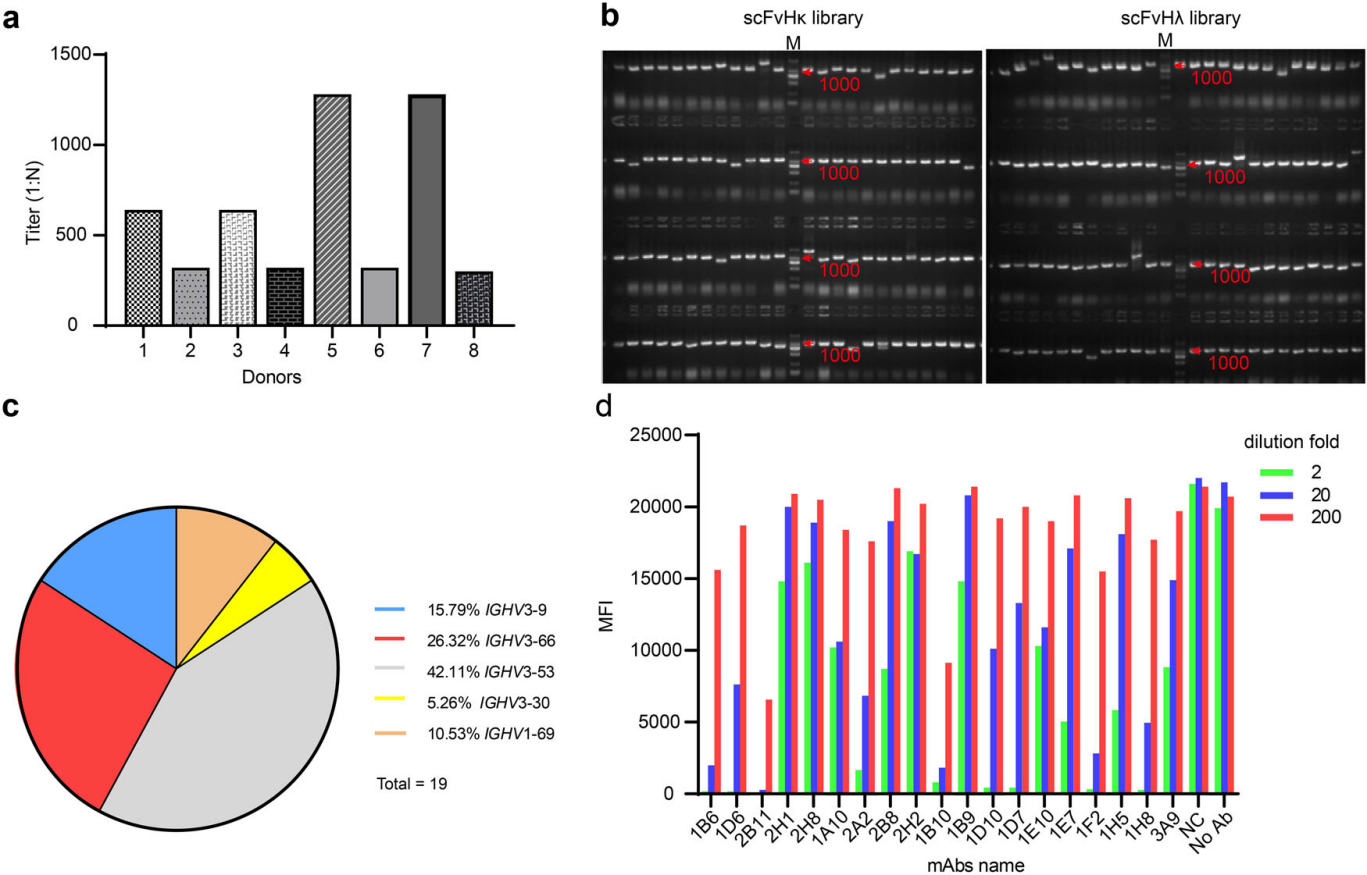
Isolation of SARS-CoV-2 S1 or RBD-binding antibodies. **a** The RBD-specific antibody titers in the plasma of different convalescent patients. The titers were represented as 1:N, and N is the highest dilution factor of a serum sample that causes a positive test reaction. **b** The insertion rate of scFvHκ and scFvH_λ_ libraries by colony-PCR. Ninety-six colonies were randomly picked for PCR, and the target fragment is about 1 kb. The DL 2000 maker was used to define the molecular weight. **c** *IGHV* gene usage assessment. The distribution of gene usage is shown for a total of 19 unique clones. **d** Competition flow cytometry assay using ACE2-expressing cells. Phage clones’ supernatants were diluted 2-, 20-, and 200-fold before testing.

In order to construct a phage-display library, we collected peripheral blood from eight COVID-19 convalescent patients after they have provided written informed consent; the RBD-specific antibody titers were then determined in each serum sample by enzyme-linked immunosorbent assay (ELISA). As expected, donors exhibited different titers of RBD-specific antibodies (Fig. 1a): two individuals showed titers over 1000, another two showed titers between 500 and 1000, and the remaining four showed titers between 200 and 500. In parallel, PBMCs were isolated and pooled for the construction of two phage-display immune libraries.

### Construction of the phage-display immune libraries, bio-panning, and validation

The total mRNA was isolated from the pooled PBMCs and converted into cDNA, and the sequences encoding the variable region of the heavy chain (VH) and two types of the variable region of the light-chain (VL_κ_, and VL_λ_) antibody regions were polymerase chain reaction (PCR)-amplified, separately, and subjected to form the single-chain fragment variable (scFv). Then, two phage-display libraries, the scFvHκ, and scFvH_λ_ libraries, with sizes of 1.37 × 10^9^ and 2.40 × 10^9^ pfu/mL, respectively (Table 1), were constructed; the VH regions were fused to the VL_κ_ or VL_λ_ regions through a linker. The quality of the two libraries was confirmed by reverse transcription PCR (RT-PCR) and phage-display ELISA, with insertion rates of over 89% (Fig. 1b), phage-display rates of over 65%, and scFv expression rates of over 77% for both libraries (Table 1). Following phage amplification, the phage titers were calculated via gradient dilution. The phage titers of scFvHκ and scFvH_λ_ were 5.00 × 10^12^ pfu/mL and 4.50 × 10^12^ pfu/mL, respectively (Table 1). Overall, the results suggest that the two scFv libraries were constructed with high quality.

**Table 1:** Characteristics of the constructed phage-display libraries.

Library	Library size	Insert rate	Correct Rate	Display rate	Expression rate	Phage titer (pfu/mL)
VHκ	1.37 × 10^9^	89.58%	77.01%	72.72%	85.23%	5.0 × 10^12^
VHλ	2.40 × 10^9^	90.63%	73.33%	65.91%	77.27%	4.5 × 10^12^

To isolate potential hits from the phage-display libraries, the SARS-CoV-2 S1 subunit, and its RBD (both recombinant), were used as the baits in three rounds of bio-panning; a total of thirteen 96-well plates were picked and screened. Remarkably, we obtained 433 hits, 217 specifically binding to the S1 subunit, and the remaining 216 specifically binding to the RBD. After sequencing, 20 phage clones containing unique sequences were obtained; binding validation was then performed by ELISA. The results demonstrated that while one antibody bound exclusively to the S1 subunit, the other 19 clones bound to both the S1 and RBD baits.

Next, with respect to the 19 clones able to bind to the RBD, we investigated the immunoglobulin heavy-chain gene-variable region (*IGHV*) gene usage (Fig. 1c and Table 2). We found that *IGHV*3-66 and *IGHV3*-53 were the most frequently used genes, including clones 2B11 and 1D7, both associated with a shorter heavy-chain complementarity-determining region 3 (HCDR3) (9–12 amino acids) and previously reported in association with anti-RBD nAbs^12,26–28^. Interestingly, in this study, these two clones showed one to three mutations in the heavy-chain variable region, while clone 1E10 showed a low mutation rate compared to *IGHV*1-69-derived antibodies, but contained a longer HCDR3 (19 amino acids). Altogether, our results showing that the sequences of nAbs isolated from COVID-19 convalescent patients are remarkably close to the germline segments, without extensive somatic hypermutations, are in line with previous observations^29^.

**Table 2:** Summary of the 19 unique clones identified.

Number	mAbs ID	Competition FACS	Clones selected	Group	Heavy chain			Light chain		
					Germline	CDR3 lengths	%SHM	Germline	CDR3 lengths	%SHM
1	2H1	★★		1	*IGHV*3-9*01	19	2.7	*IGLV*2-14*01	10	0.7
2	1A10	★★★		1	*IGHV*3-9*01	19	2.7	*IGLV*2-14*01	10	0.7
3	3A9	★★★	▲	1	*IGHV*3-9*01	19	2.4	*IGLV*2-14*01	10	1.4
4	1F2	★★★★	▲	2	*IGHV*3-66*01	12	1.4	*IGKV*3-20*01	9	0.7
5	1D10	★★★★	▲	3	*IGHV*3-53*04	12	2	*IGKV*1-12*01	8	0.7
6	1H5	★★★		3	*IGHV*3-53*04	12	2.1	*IGKV*1-9*01	9	1.8
7	1B6	★★★★	▲	4	*IGHV*3-66*01	12	2.7	*IGLV*3-1*01	9	6.1
8	1B9	★★		4	*IGHV*3-53*02	12	2.4	*IGKV*1-27*01	9	2.8
9	1H8	★★★★	▲	4	*IGHV*3-66*01	12	2.7	*IGKV*1-27*01	9	2.1
10	1D6	★★★★		4	*IGHV*3-53*02	12	2.4	*IGLV*3-21*03	11	0
11	2B11	★★★★	▲	4	*IGHV*3-66*01	12	2.7	*IGLV*1-44*01	12	8.4
12	2B8	★★★		4	*IGHV*3-66*01	12	2.4	*IGLV*1-44*01	12	11.5
13	1E7	★★★★		5	*IGHV*3-53*01	11	0.7	*IGKV*1-12*01	10	0.4
14	1D7	★★★★	▲	5	*IGHV*3-53*01	11	1	*IGKV*1-39*01	11	0.4
15	2A2	★★★★	▲	5	*IGHV*3-53*01	11	1.4	*IGLV*2-14*01	10	0.7
16	1E10	★★★	▲	6	*IGHV*1-69*04	19	0.3	*IGKV*1-39*01	9	0.4
17	2H8	★		7	*IGHV*3-30*18	19	1.4	*IGLV*3-21*02	11	1.8
18	2H2	★		8	*IGHV*1-69*10	17	0.7	*IGLV*1-40*01	11	2.3
19	1B10	★★★★	▲	9	*IGHV*3-53*02	11	1	*IGLV*2-8*01	10	2.7

*IGHV* the immunoglobulin heavy-chain gene-variable region, *IGLV* the immunoglobulin lambda light-chain gene-variable region, *IGKV* the immunoglobulin kappa light-chain gene-variable region.

Five ★ represent for clones with strong competition. The triangle-marked (▲) clones were chosen for IgG conversion, based on their competition and diversity.

Finally, to understand the potential of the selected clones to block the interaction between the RBD and hACE2, we performed a competition fluorescence-activated cell sorting (FACS)-based assay using hACE2-expressing cells (Fig. 1d). The results revealed that the 19 phage candidates showed distinct blocking activities in the context of hACE2-expressing cells; finally, ten clones were selected based on both the competition effects and *IGHV* gene diversity for further analysis (Table 2).

### Generation, expression, and purification of full-length monoclonal antibodies

The ten pairs of scFV genes were amplified by PCR based on the above-selected phage clones and fused with a constant region of human immunoglobulin G1 (IgG1). Monoclonal antibodies (mAbs) were then expressed in Expi293F cells after transfection with the generated constructs. Afterward, the obtained antibodies were purified from the cell culture supernatants via protein A affinity chromatography and subjected to sodium dodecyl sulfate-polyacrylamide gel electrophoresis (SDS-PAGE). Importantly, the purity of all antibodies reached 95% after size-exclusion chromatography, and two bands with 50 kDa and 25 kDa were observed after SDS-PAGE under reducing conditions, corresponding to the IgG heavy and light chains, respectively. Overall, these results suggest that purified mAbs were successfully obtained.

### Binding and blocking ability of the anti-SARS-CoV-2 S1 (RBD) antibodies

**Fig. 2:**
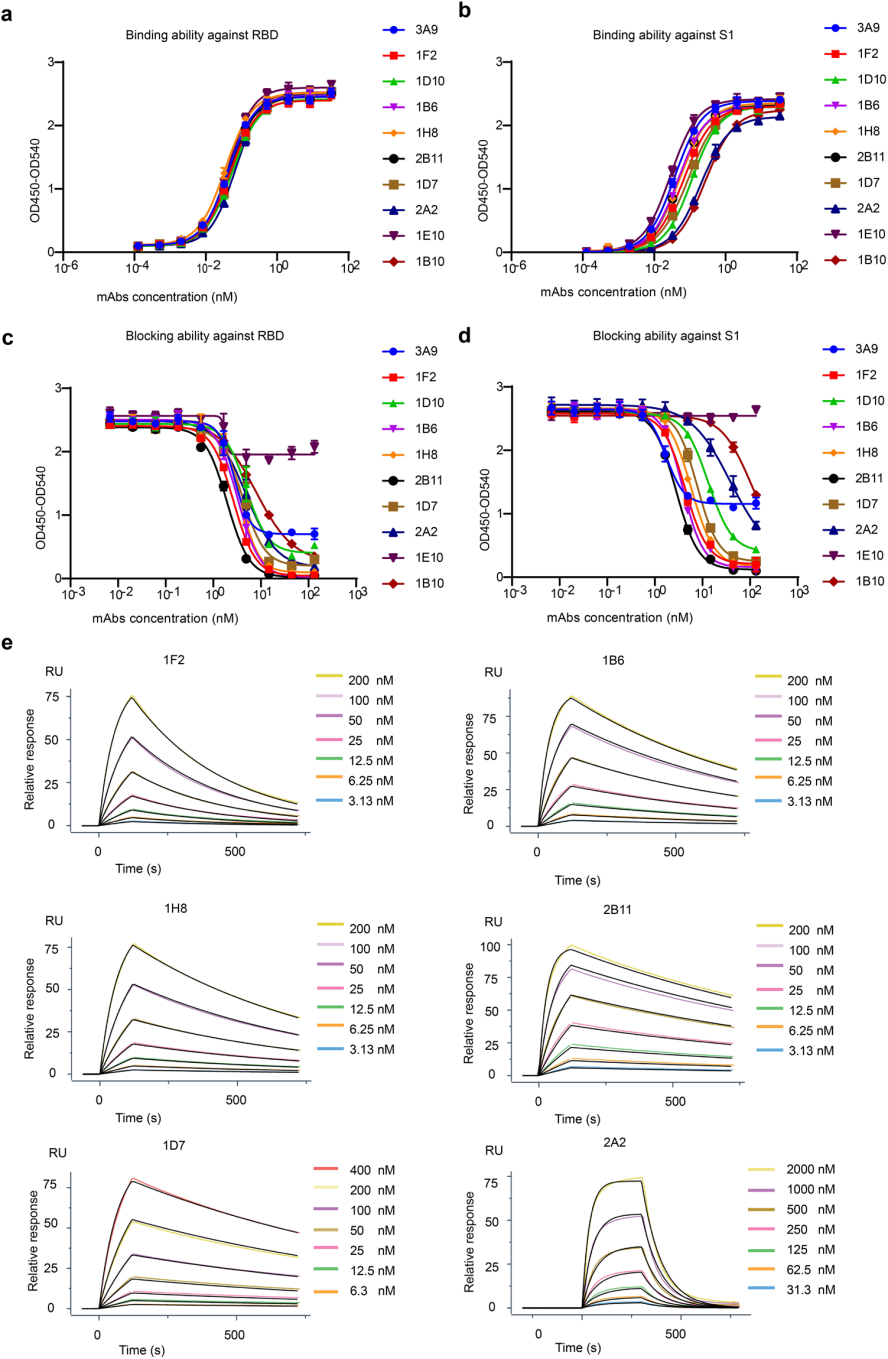
Identification of SARS-CoV-2-neutralizing mAbs. The binding ability of the antibodies to RBD (**a**) and S1 (**b**) was measured via binding ELISA. The blocking ability of antibodies toward RBD (**c**) and S1 (**d**) was measured via competition ELISA. **e** The dissociation constants of the representative mAbs against the S1 protein were determined using SPR. K_D_ was calculated using a 1:1 binding model.

To determine the binding ability of the obtained mAbs, we calculated the 50% effective concentration (EC_50_) using binding ELISA analysis (Fig. 2a, b and Table 3). Six out of the ten mAbs showed strong binding affinities to both the RBD and the S1 subunit, with EC_50_ values lower than 0.07 nM. Of note, except for clones 3A9 and 1E10, most mAbs showed higher binding affinity (lower EC_50_ values) to the RBD than to the S1 subunit. Next, we performed competitive ELISA assays and calculated the half-maximal inhibitory concentration (IC_50_) (Fig. 2c, d and Table 3). We found that among the ten mAbs, seven exhibited the ability to block the binding between ACE2 and both S1 and RBD sequences, while two (2A2 and 1B10) could only block the interaction between ACE2 and RBD; interestingly, although the remaining antibody (1E10) showed relatively strong binding affinities to both S1 and RBD (EC_50_ = 0.029 nM and 0.044 nM, respectively), no blocking ability was detected. Notably, clone 2B11 exhibited the highest blocking potential (IC_50_ = 1.96 nM for RBD and IC_50_ = 2.79 nM for S1); the maximum inhibition rate of the binding between RBD and hACE2 exceeded 99%. Therefore, these results suggest that the determination of the binding activity to S1 or RBD in the context of anti-SARS-CoV-2 mAbs may not be enough to predict their ability to block RBD–hACE2 interactions. Surface plasmon resonance (SPR) experiments were also carried out for the calculation of the kinetic rate and affinity constants of the mAbs (Fig. 2e and Table 3), revealing that three clones exhibited the equilibrium dissociation constant (K_D_) values lower than 20 nM; of note, clone 2B11 showed the lowest K_D_ (4.67 nM).

**Table 3:** Summary of ten selected antibodies.

mAbs	Predicted epitope	Binding EC_50_ (nM)		KD (nM)	Blocking IC_50_ (nM)				Pseudovirus neutralization IC_50_ (ng/mL)	Authentic virus PRNT IC_50_ (ng/mL)
		S1	RBD		S1	Inhibition max (%)	RBD	Inhibition max (%)		
3A9	RBD-2	0.038	0.042	N.D.	1.74	57	2.76	72	93	1541
1F2	RBD-1	0.068	0.048	39.4	4.34	92	2.65	98	40	25
1D10	RBD-1	0.115	0.054	N.D.	13.32	85	4.91	84	73	85
1B6	RBD-1	0.047	0.043	11.3	3.88	94	3.25	99	12	18
1H8	RBD-1	0.054	0.035	18.7	5.76	94	3.6	96	20	35
2B11	RBD-1	0.051	0.047	4.76	2.79	95	1.96	99	6	1
1D7	RBD-1	0.092	0.055	24.2	7.8	91	4.1	92	30	34
2A2	RBD-1	0.201	0.067	1070	N.A.	N.A.	4.85	93	705	1178
1E10	RBD-2	0.029	0.044	N.D.	N.A.	N.A.	N.A.	N.A.	94	21
1B10	RBD-1	0.27	0.053	N.D.	N.A.	N.A.	7.83	90	112	79

*K*_*D*_ dissociation constant at equilibrium, *N.D.* not detectable, *N.A.* no appreciable.

Epitope were predicted by Epitope binning via bio-layer interferometry. EC_50_ and IC_50_ were calculated by fitting to a four-parameter logistic curve with GraphPad Prism Version 8.0.1.

### Bio-layer interferometry competition binning analysis

To bin the epitopes recognized by the ten antibodies, we used the bio-layer interferometry technology (Table 4). The results showed that eight antibodies, including the 2B11, 1B6, 1H8, and 1D7 clones derived from the *IGHV*3-66 and *IGHV*3-53 germline heavy chains recognized the same (or similar) epitope(s) on RBD (epitope 1), while the other two antibodies, clones 1E10 (*IGHV*1-69-derived) and 3A9 (HV3-9-derived) interacted with different RBD epitopes (epitope 2). Importantly, these results were consistent with the ELISA-binding/blocking activity ones, showing that, although both 1E10 and 3A9 clones bound to S1 and RBD, 1E10 did not block RBD–hACE2 interactions, and 3A9 partially block RBD–hACE2 interactions. Altogether, our results indicate that 1E10 binds to a region of RBD outside the hACE2-interacting site, the top of the trimeric S protein of SARS-CoV-2^1,4^.

**Table 4:** Epitope binning of the ten selected neutralizing mAbs through bio-layer interferometry.

1st^a^ 2nd^a^	2A2	1B6	1F2	2B11	1D10	1H8	1D7	1B10	1E10	3A9
2A2	4.33%	7.78%	6.29%	8.86%	7.71%	4.55%	8.41%	17.79%	**93.62%**	**90.31%**
1B6	5.15%	6.29%	6.23%	7.56%	11.24%	4.52%	8.58%	9.03%	**102.46%**	**101.53%**
1F2	7.04%	8.68%	7.31%	9.61%	10.57%	6.70%	11.56%	12.86%	**99.48%**	**102.76%**
2B11	1.67%	5.97%	5.79%	5.37%	7.33%	3.46%	8.48%	7.19%	**104.27%**	**104.70%**
1D10	9.32%	14.45%	13.15%	14.04%	13.10%	11.82%	14.15%	17.09%	**100.40%**	**99.83%**
1H8	7.87%	12.61%	12.86%	12.51%	12.64%	11.47%	14.54%	15.11%	**103.32%**	**105.11%**
1D7	2.66%	6.37%	5.50%	5.32%	7.01%	6.02%	7.62%	7.61%	**104.69%**	**106.59%**
1B10	2.82%	0.70%	2.47%	1.62%	7.68%	1.74%	5.29%	4.12%	**87.59%**	**89.10%**
1E10	**107.24%**	**106.24%**	**105.42%**	**101.30%**	**109.57%**	**106.27%**	**107.48%**	**95.64%**	6.69%	5.26%
3A9	**103.89%**	**104.83%**	**103.02%**	**99.69%**	**105.96%**	**99.13%**	**101.98%**	**97.83%**	11.38%	6.03%

^a^The column and the row were shown for the primary and secondary antibodies. Antibodies bound to the same or similar epitope are shown in the same font (unbold and bold).

### Neutralization potency against WT SARS-CoV-2

**Fig. 3:**
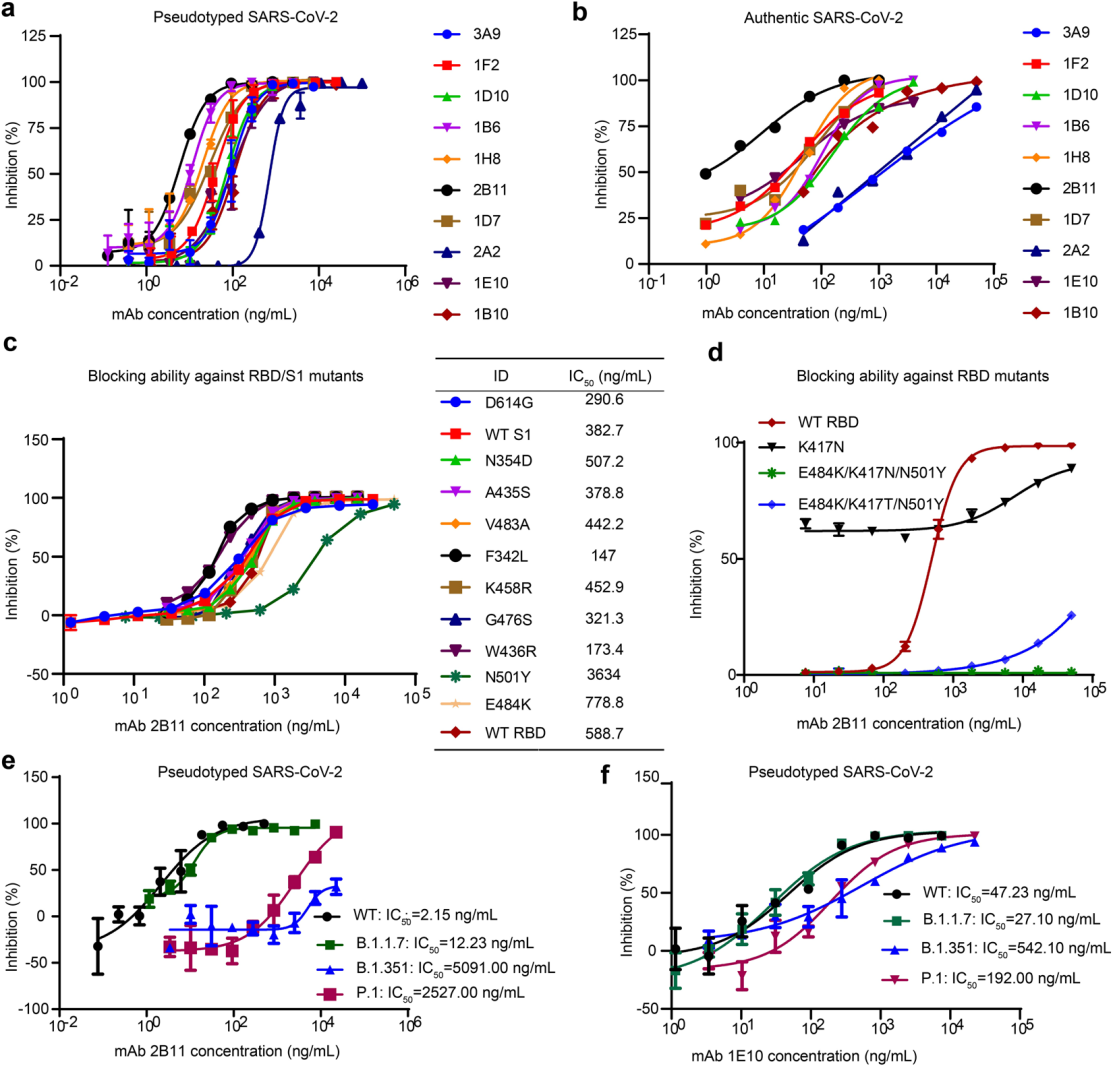
Characteristics of the neutralization activity of the selected antibodies against WT SARS-CoV-2 and its emerging variants. The SARS-CoV-2-neutralizing activity of ten selected antibodies against pseudoviruses (**a**) and authentic viruses (**b**). Blocking activity of 2B11 against RBD/S1 single-point mutant proteins (**c**) and multipoint mutant proteins (**d**). Neutralization ability of the 2B11 (**e**) and 1E10 (**f**) mAbs against three newly dominant SARS-CoV-2 variants. WT refers to the original, non-mutated protein or virus.

To evaluate the neutralization potency against WT SARS-CoV-2 of the mAbs, we conducted an in vitro SARS-CoV-2 neutralization assay using both SARS-CoV-2 pseudoviruses and authentic viruses (Fig. 3a, b and Table 3). The IC_50_ results for pseudoviruses and authentic viruses were comparable; half of the mAbs showed IC_50_ values lower than 50 ng/mL. Notably, 2B11 showed the most potent neutralization ability, with IC_50_ = 6 ng/mL for pseudovirus and IC_50_ = 1 ng/mL for authentic viruses. Interestingly, 1E10, targeting another epitope, also showed highly neutralization potency, with IC_50_ = 21 ng/mL for authentic viruses. Importantly, the neutralization ability of the eight mAbs (targeting the hACE2-binding epitope, epitope 1) was basically consistent with their blocking ability.

### Blocking and neutralizing ability against mutant SARS-CoV-2 variants

Many mutations of SARS-CoV-2 have been found in nature, and some are associated with immune escape in vivo. Recently, the SARS-CoV-2 variants B.1.1.7 (bearing mutations, including N501Y/D614G), B.1.351 (bearing mutations, including K417N/E484K/N501Y/D614G), and P.1 (bearing mutations, including K417T/E484K/N501Y/D614G) have rapidly become the dominant variants in South Africa, UK, and Brazil, respectively. Therefore, determining whether a mAb can effectively neutralize these mutant strains is critical to prioritizing the subsequent clinical development. First, we tested the blocking ability of 2B11, the most promising nAb, against a panel of RBD and S1 mutants, including those with the single-point mutations D614G, N501Y, E484K, K417N, and those with the multipoint mutations E484K/K417N/N501Y and E484K/K417T/N501Y. The results showed that the blocking ability of 2B11 was slightly increased or decreased by most of the single-point mutations including D614G and E484K (Fig. 3c). However, the blocking potential of 2B11 was largely reduced in the context of the K417N and N501Y single-point mutation (Fig. 3c, d). Moreover, the blocking ability of 2B11 was almost completely lost in the context of the multipoint E484K/K417N/N501Y and E484K/K417T/N501Y mutations (vs WT proteins; Fig. 3d). We also tested the binding ability between 2B11 or ACE2 and mutant proteins, including K417N, E484K, N501Y, E484K/K417N/N501Y, and E484K/K417T/N501Y, and found that the binding ability of 2B11 against E484K/K417T/N501Y and E484K/K417N/N501Y was completely lost, and against K417N were largely reduced, while against E484K and N501Y was unaffected (Supplementary Fig. S1a–c). Moreover, N501Y mutation was reported with increased affinity to ACE2^30^, which was the reason for the reduced blocking ability of 2B11, while K417N reportedly displayed reduced affinity to ACE2 (Supplementary Fig. S1d, e).

As an alternative approach, we further assessed the neutralization activity of 2B11 against pseudoviruses bearing a full package of B.1.1.7, B.1.351, or P.1 mutations. For the sake of comparison, we used 1E10 antibodies, with a different RBD-binding region, in the same assay, and the results are displayed in Fig. 3e, f for 2B11 and 1E10, respectively. We found that the neutralizing ability of 2B11 against B.1.351 and P.1 pseudoviruses was significantly decreased, while it still exhibited potent neutralizing ability against B.1.1.7 pseudoviruses, though slightly impaired compared with the WT counterparts (single-digit fold in the IC_50_ neutralization titers). Meanwhile, 1E10 antibodies completely neutralized the three mutant pseudoviruses with different potencies. We found that B.1.351 was the most resistant to neutralization by 1E10 (IC_50_ = 0.542 μg/mL), followed by P.1, while the neutralization of B.1.1.7 was slightly increased.

### Structure of 2B11-Fab in complex with RBD

**Fig. 4:**
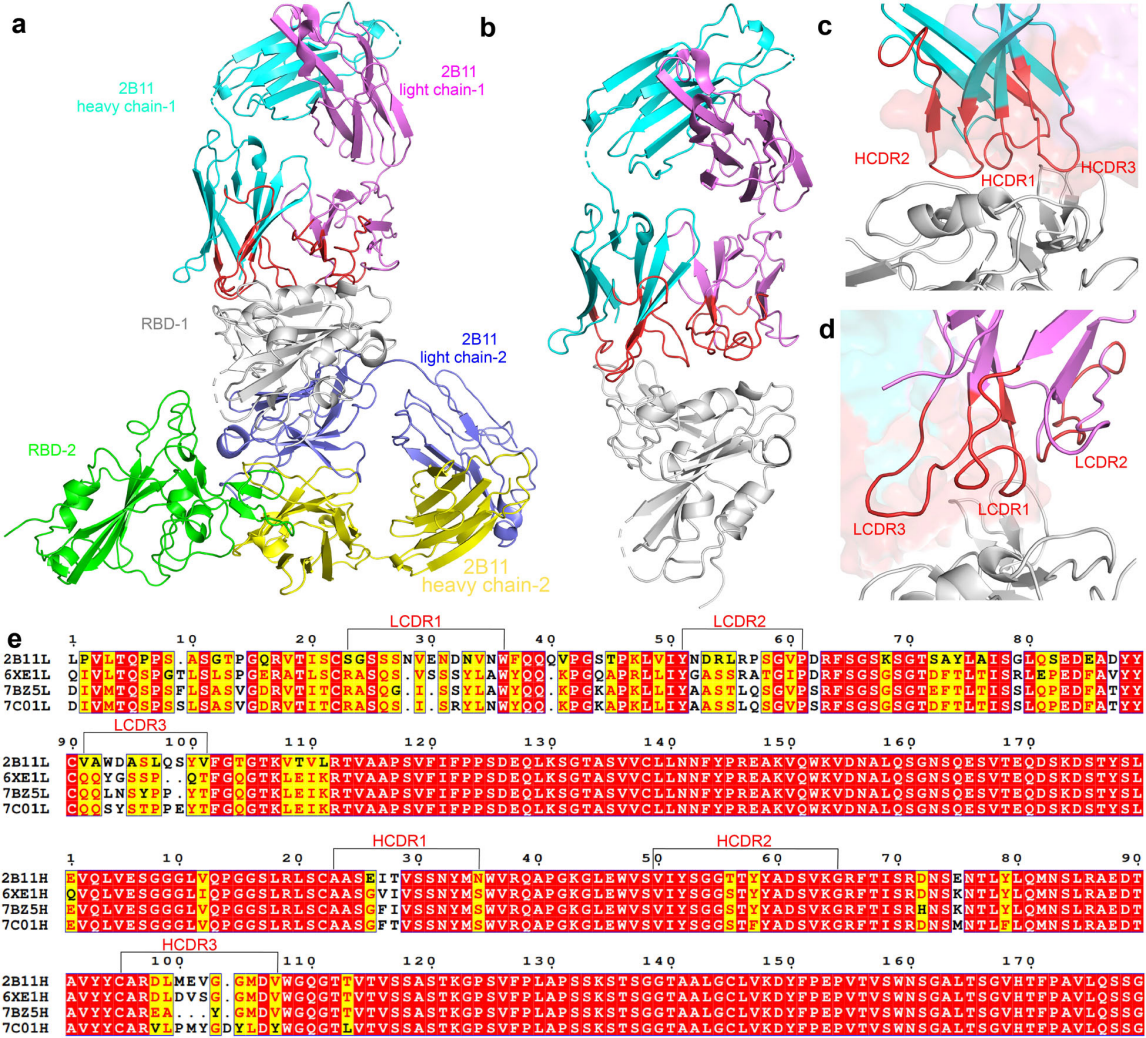
Overall structure of the 2B11-Fab in complex with the SARS-CoV-2 RBD. **a**, **b** The overall structure of 2B11-Fab in complex with the SARS-CoV-2 RBD. The structure is shown as a cartoon. The 2B11 heavy chain is shown in cyan and yellow. The light chain is shown in violet and light blue. RBD is shown in gray and green. The CDRs are shown in red. **c**, **d** Details of the interactions between the 2B11 heavy (up) and light (down) chains and the RBD. The CDRs are color-coded. **e** Sequence alignment of the 2B11 heavy and light chains with those of reported Fab complexes. The black brackets above the sequence highlight the residues that interact with the RBD.

Next, to understand the mechanism behind the highly potent SARS-CoV-2 neutralization ability of 2B11, we solved the crystal structure of the 2B11-Fab in complex with RBD at a resolution of 3.59 Å. We observed two 2B11-Fab–RBD complexes in an asymmetric unit in the crystal structure, stacked in an inverted T-shape (Fig. 4a); this may explain why the crystals of 2B11-Fab–RBD are difficult to stack and easily form lamellar twins. In addition, although the obtained resolution is not sufficient to fully reveal the details of the molecular interactions between 2B11-Fab and RBD, it is enough to confirm that the CDR loops of 2B11 overlap with the ACE2-binding epitope of the RBD with a tight interaction interface area of ~978 Å^2^ (Fig. 4b). Importantly, our results show that the heavy chain plays an important role in the binding to RBD, contributing with ~739 Å^2^ of the interaction (Fig. 4c, d). In addition, sequence alignment showed that the heavy chain of mAb 2B11 was similar to those of other previously reported SARS-CoV-2 nAbs including CV30^31^, B38^32^, and CB6^33^ (PDB: 6XE1^31^, 7BZ5^32^, and 7C01^33^), indicating that 2B11 originates from *IGHV*3-53/*IGHV*3-66 germline segments (Fig. 4e). However, the relatively low conservation of light chains indicates that they originate from different germlines, implying that *IGHV*3-53/*IGHV*3-66 could pair with different light chains to target the ACE2-binding site of the SARS-CoV-2 RBD. Altogether, the above results indicate that the heavy chain from the *IGHV*3-53 or *IGHV*3-66 germline plays a leading role in the process of RBD recognition.

**Fig. 5:**
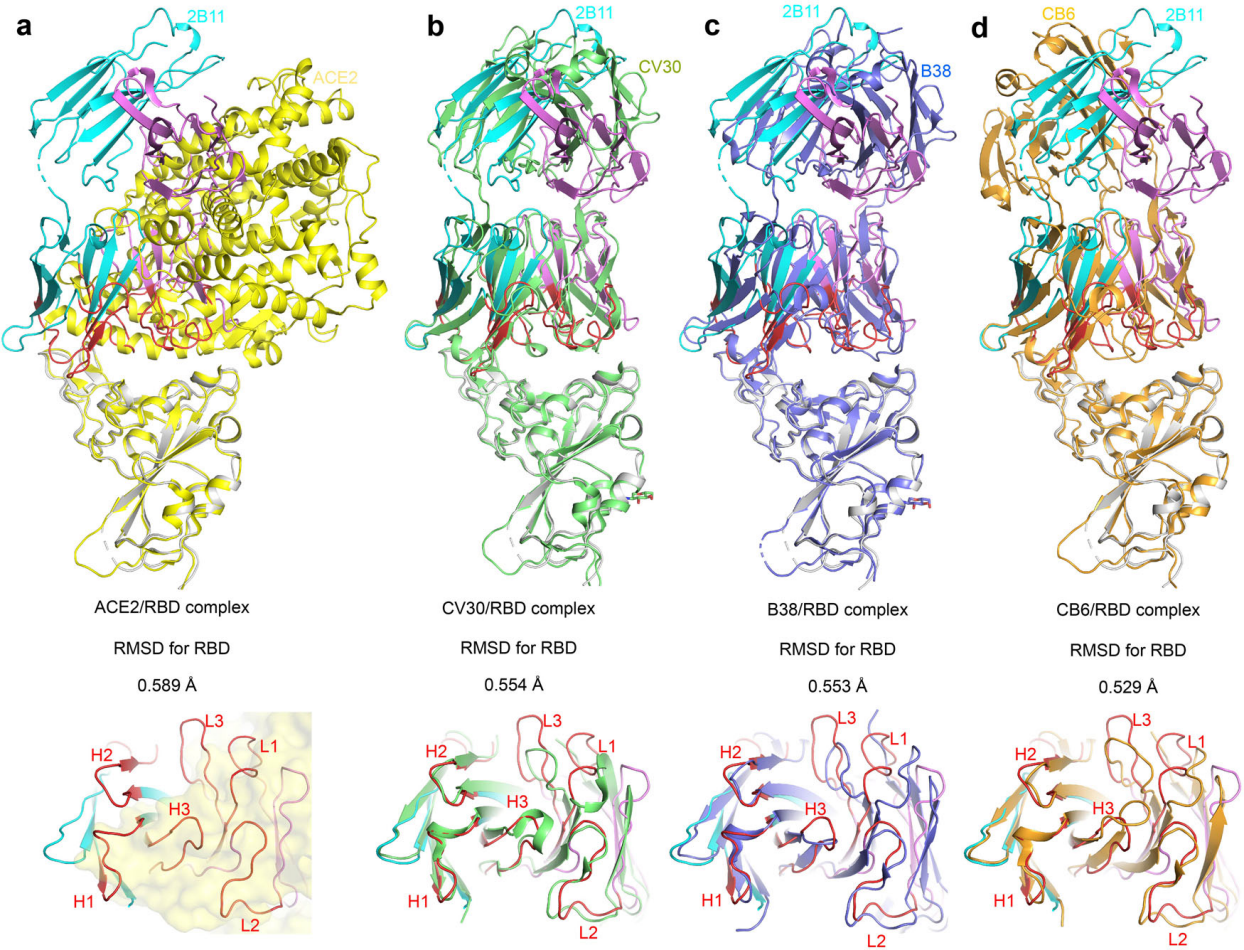
Comparison of the complexes between RBD and ACE2, or the 2B11-Fab, or other *IGHV*3-53/*IGHV*3-66 germline-derived neutralizing antibodies. **a** Structural alignment between the ACE2/RBD and 2B11/RBD complexes. The ACE2/RBD complex is shown in yellow. The image below is the bottom view of the main image without the RBD. **b** Structural alignment between the 2B11/RBD and CV30/RBD complexes. The CV30/RBD complex is shown in green. **c** Structural alignment between the 2B11/RBD and B38/RBD complexes. The B38/RBD complex is shown in light blue. **d** Structural alignment between the 2B11/RBD and CB6/RBD complexes. The CB6/RBD complex is shown in orange.

Importantly, the conformation of RBD in the 2B11-Fab-RBD complexes is similar to that reported in different complexes (Fig. 5a–d), including a complex between the SARS-CoV-2 RBD and ACE2 (PDB: 6M0J;^34^ Fig. 5a), and SARS-CoV-2 RBD-CB6 complexes (PDB: 7C01;^3331^ Fig. 5d). In fact, although the conformation of 2B11-Fab CDR loops is similar to that of CV30, B38 and CB6 mAbs, there are some differences in the detailed conformation of the interaction interface (Fig. 5b–d, top). Further, the structural alignment results showed that the structural conformation of the HCDR was more consistent than that of the light chain (Fig. 5b–d, bottom). Of note, HCDR1 and HCDR2 interact with residues 455–491 in the long RBD loop (Fig. 4c), while CDR1 and CDR3 in the light chain interact mainly with residues 403–411 and residues 497–506 (Fig. 4d). Overall, these results suggest that the potent neutralization activity of 2B11 mAbs mainly depends on the direct interface-residue competition with hACE2 and the consequent blocking of the interaction between RBD and hACE2.

**Fig. 6:**
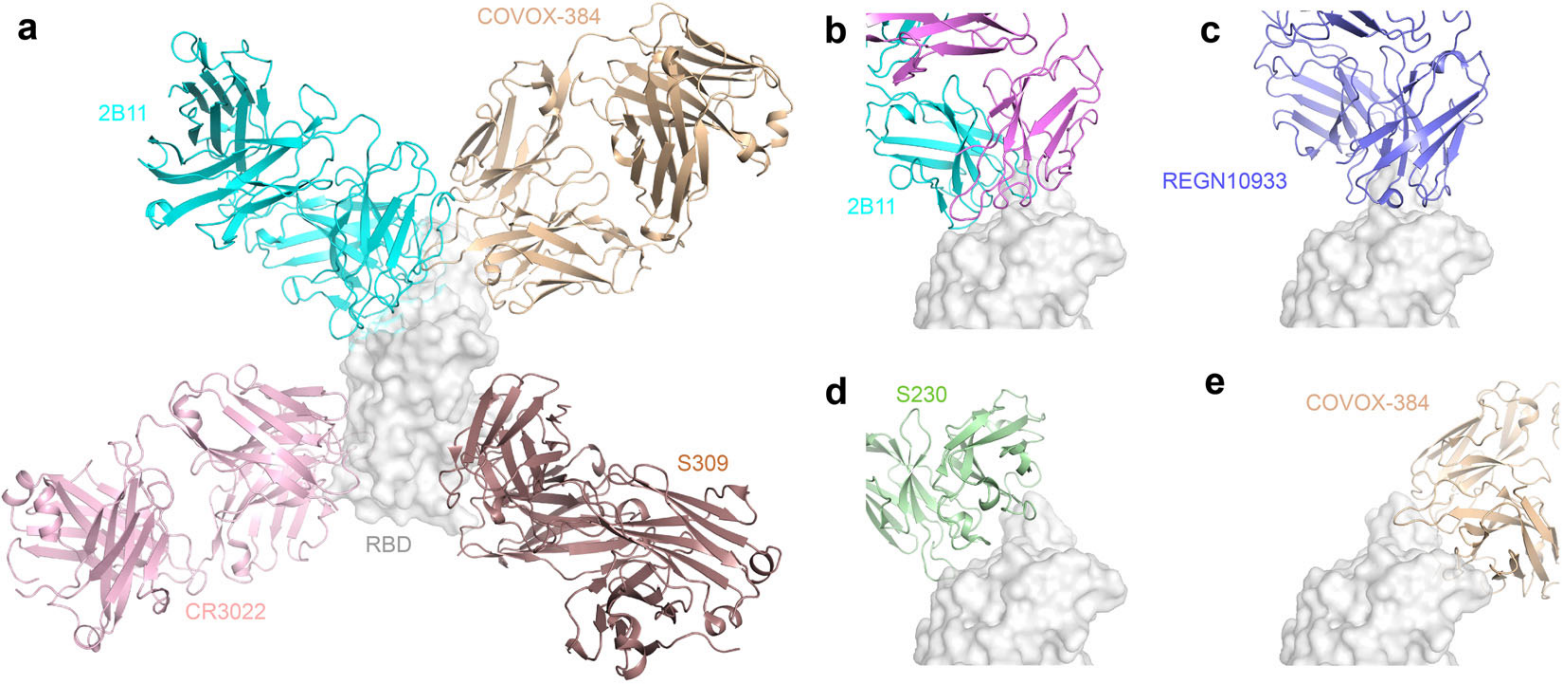
Comparison of the complexes between RBD and the 2B11-Fab, or other different *IGHV* germline-derived neutralizing antibodies. **a** The 2B11, S309, CR3022, and COVOX-384 mAbs recognize different RBD epitopes. The RBD is shown in gray; 2B11, S309, CR3022, and COVOX-384 are shown in cyan, brown, pink and malt, respectively. **b**–**e** The 2B11 (**b**), REGN10933 (**c**), S230 (**d**), and COVOX-384 (**e**) bind to different sites of the RBD ridge.

In addition, the structural comparison of available antibodies from different *IGHV* germline gene sources showed that most of the antibodies bind to the RBD ridge of the S protein (Fig. 6a–e); however, some antibodies were found to target other regions of RBD and did not compete with ACE2. For example, S309 binds to the epitope near the early glycosylation site^35^, and CR3022 binds to the key region for the up-down conformation switching of RBD^36^ (Fig. 6a). In addition, 2B11, COVOX-384^35^, S230^37^, and REGN10933^14^ all compete with ACE2 via binding to the RBD ridge, with neutralizing activity. However, while the binding sites of 2B11 and REGN10933 are mainly located at the tip of the ridge with a larger binding surface area for 2B11 (Fig. 6b, c), those of S230 and COVOX-384 are located on both sides of the ridge (Fig. 6d, e).

### Prophylactic and therapeutic potential of 2B11 mAbs in vivo

**Fig. 7:**
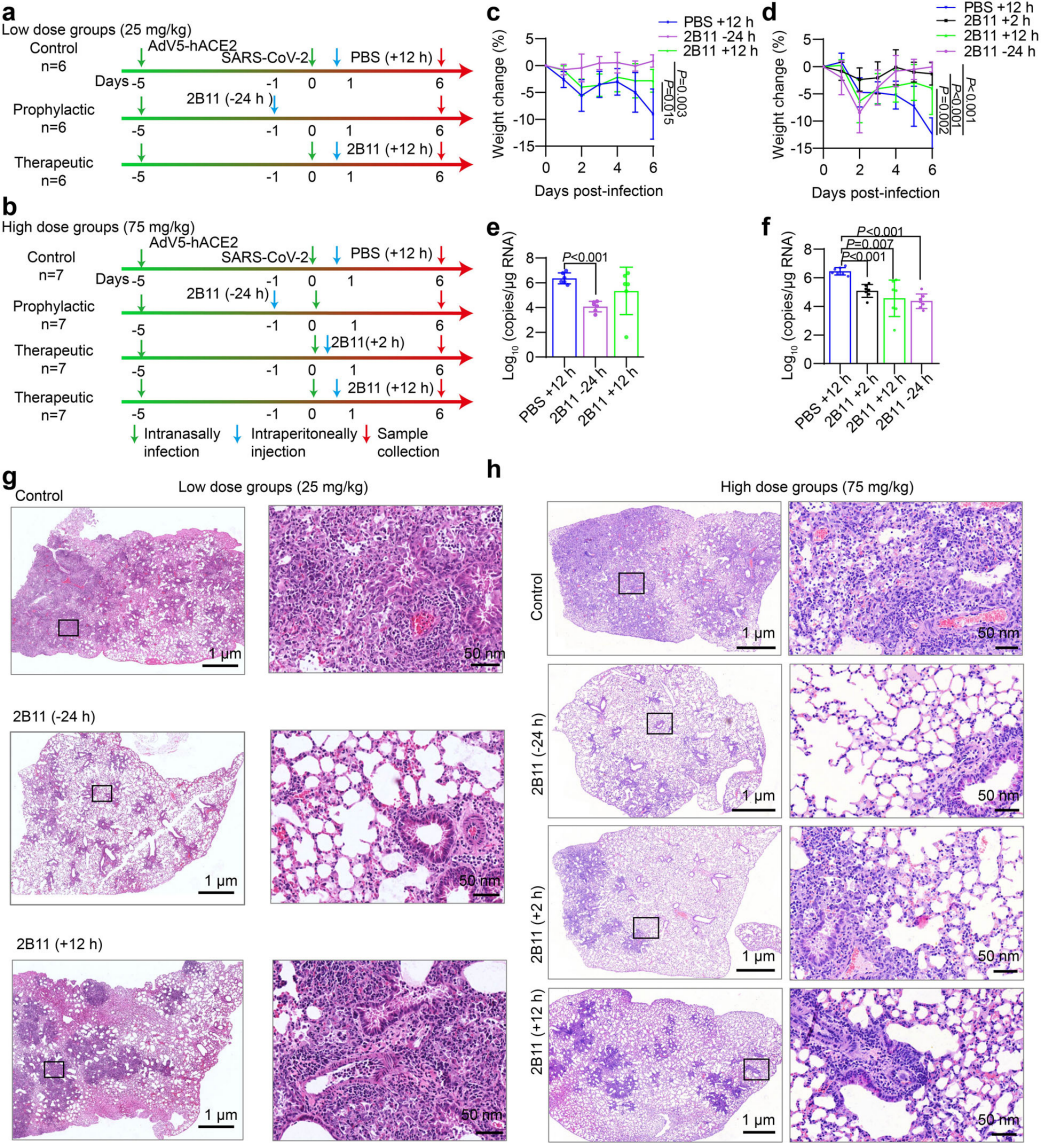
The mAb 2B11 shows therapeutic and prophylactic potential in SARS-CoV-2-infected AdV5-hACE2-transduced mice. **a**, **b** The experimental design. Two independent experiments were performed. **a** In the low-dose experiment, mice were intraperitoneally injected with 25 mg/kg of 2B11 24 h before (–24 h) or at 12 h after (+12 h) SARS-CoV-2 infection; control mice were injected with 200 μL PBS at +12 h (*n* = 6 per group). **b** In the high-dose experiment, the mice were treated with PBS 2 h after or 75 mg/kg of 2B11 24 h prior, or 2 h, or 12 h after infection (*n* = 7 per group). **c**, **d** The body weight changes in the low- (**c**) and high-dose (**d**) 2B11-treated hACE2-transduced mice challenged with SARS-CoV-2 were recorded over 6 days. **e**, **f** The viral RNA levels in the lung tissues of the low- (**e**) and high-dose (**f**) 2B11-treated mice, were determined using qRT-PCR at 6 days post infection. Data are represented as the mean ± SD. Statistical significance was determined using the two-tailed Student’s *t-*test; *P* values are represented (of note, the *P* value between PBS + 12 h and 2B11 + 12 h in **e** is 0.233). **g**, **h** Histopathological analyses of 2B11-treated or untreated mice infected with SARS-CoV-2. Representative images of lung sections stained with hematoxylin and eosin in the context of low-dose (**g**) and high-dose (**h**) experiments (6 days post challenge).

Finally, to evaluate the prophylactic and therapeutic potential of 2B11 mAbs against SARS-CoV-2 infection in vivo, AdV5-hACE2-transduced IFNAR^–/–^ mice, highly susceptible to SARS-CoV-2 infection (typically with severe lung pathology and high lung virus titers)^38,39^, were used in two independent experiments, as illustrated in Fig. 7a, b. Briefly, the mice were challenged with SARS-CoV-2 via the intranasal route 5 days after the intranasal transduction with AdV5-hACE2, and their body weights were monitored over a 6-day time course and the viral loads in the lungs were detected by the qRT-PCR at day 6 post-infection. In the low-dose experiment (Fig. 7a), mice were injected with PBS (as a control group) or 25 mg/kg of 2B11, 24 h before (prophylactic treatment, –24 h) or 12 h after SARS-CoV-2 challenge (treatment, +12 h); in the high-dose experiment (Fig. 7b), animals received PBS or 75 mg/kg of 2B11 24 h before (–24 h prophylaxis group), or 2 or 12 h after (+2 h and +12 h therapeutic groups) SARS-CoV-2 challenge. Compared to the PBS treatment group, all of the 2B11 prophylaxis or therapeutic groups showed a significantly reduced degree of SARS-CoV-2-induced body weight loss at 6 days post infection (*P* < 0.05, Fig. 7c, d). These results indicate that the administration of 2B11 mAbs could improve the physiological condition of SARS-CoV-2-infected mice both pre- and post infection. In addition, we also found that the load of SARS-CoV-2 was significantly reduced in the lungs of all prophylactic groups as well as in the high-dose therapeutic groups, compared to that in control animals (*P* < 0.05, Fig. 7e, f). For the high-dose therapeutic approach, we found a lower degree of body weight loss and lower viral load was associated with earlier 2B11 intervention (+2 h vs +12 h) but was not statistically significant (*P* = 0.214 and *P* = 0.343, respectively). Moreover, in mice pre-treated with either 25 or 75 mg/kg of 2B11 mAbs, only mild lung damage was observed at 6 days post challenge; most alveolar septa and cavities were normal and only small amounts of immune cell infiltrate around the bronchi/bronchioles and blood vessels were observed (Fig. 7g, h). Similar to the prophylactic group, mice treated with 75 mg/kg of 2B11 mAbs at 2 h post challenge also manifested mild pathological changes. For mice treated with 25 mg/kg or 75 mg/kg of 2B11 mAbs at 12 h post challenge, moderate levels of diffuse alveolar damage, and moderate levels of peribronchial and perivascular cuffing and interstitial inflammation were detected (Fig. 7g, h). While the PBS-treated mice displayed severe damage in the lung, characterized as diffuse alveolar damage including thickening of alveolar septa, extensive fibrosis in the alveoli, marked epithelial hyperplasia in the bronchi/bronchioles, and extensive immune infiltration in the alveoli, and around bronchi and the vessels (Fig. 7g, h). Overall, these results obtained in the context of a sensitive mouse model suggest that 2B11 exhibited encouraging prophylactic and therapeutic effects in the context of SARS-CoV-2 infection.

## Discussion

Although the generation of fully human nAbs can be performed using transgenic mice or single B-cell sorting, the use of phage-display technologies for antibody screening still has unique advantages, including ease of use, robustness, cost-effectiveness, and high throughput. Moreover, phage-display libraries are reusable sources for the isolation of different antigen-specific mAbs. In this study, using pooled PBMCs collected from eight convalescent patients as raw materials, we constructed two phage human immune libraries with a high capacity (10^9^) to increase the probability of obtaining highly potent neutralizing antibodies. Unlike the comparable proportion of RBD-binding and NTD-binding antibodies isolated from an immune phage-display library in a previously reported study^40^, 19 out of the 20 unique clones isolated in this study were able to bind to RBD, which was consistent with the results from a recently report^41^. We speculated that the distinct panning and screening conditions might be one of the reasons for these differences: the binding affinity against S1 baits of NTD binders might be lower than that of potent RBD binders, which resulted in the fact that NTD binders were eluted in the panning and screening steps.

Among the ten selected antibodies, 2B11 showed the most potent neutralizing ability against authentic SARS-CoV-2 viruses (IC_50_ = 1 ng/mL), comparable or even better than those reported for other anti-SARS-CoV-2 antibodies^12–14,26,27^. We also found that 2B11 targets the ACE2–RBD-binding region with a highly overlapped binding epitope, similar to previously reported class 1 nAbs^42^. Moreover, the neutralization activity of 2B11 against B.1.351 or P.1 pseudovirus was significantly impaired. Although the structural analysis in this study was insufficient to fully illustrate the mechanism behind this, based on the blocking and binding abilities determined against different mutants, we speculate that it is most likely mediated by K417N/T mutation. Two recent studies^7,43^ showed that the neutralization activities of several previously found antibodies (including those already in the clinic trails) against B.1.1.7 and B.1.351 variants were reduced or completely lost, mainly due to the K417N, E484K, and N501Y mutations alone, or in combination. A proper antibody cocktail, such as combining two, or even more^44^ antibodies targeting non- or partially competitive epitopes on RBD or NTD regions, can effectively limit the generation of escape mutants and the occurrence of antibody resistance caused by mutated SARS-CoV-2 variants. As a precedent, Zmapp, which includes three distinct epitopes-targeted monoclonal antibodies, exhibited satisfactory efficacy against the Ebola virus and associated variants in Guinea^45^. REGN-COV2^14,46^, which is a cocktail of two non-competitive neutralizing antibodies (REGN10933/REGN10987), can dramatically limit the generation of escape mutants^47^, and were the first to be approved by the FDA as an emergency treatment for COVID-19. At present, several antibody cocktails such as AZD7442^48^ (COV2-2196/COV2-2130) and BRII^49^ (BRII-196 and BRII-198) are in phase 3 clinical trials, with more in different stages of the pepeline^49^. As for 2B11, its ability to neutralize original WT SARS-CoV-2 and B.1.1.7 suggests it as a promising candidate, partnering with neutralizing antibodies against other variants to be used in cocktail strategy for COVID-19 treatment.

In our report, the *IGHV*3-66 and *IGHV*3-53 germline gene segments, associated with D, and JH6 or JH4 segments (shorter HCDR3 length; 11–12 amino acids) were most frequently found in the 20 unique clones, followed by the *IGHV*3-9, *IGHV*1-69, and *IGHV*3-30 germline gene segment. In line with these results, eight of the ten selected neutralizing antibodies, including 2B11, were encoded by *IGHV*3-66 or *IGHV*3-53 germline gene segments and shared the same (or similar) epitope(s). Recently, many *IGHV*3-66 and *IGHV*3-53 germline gene segments-encoded SARS-CoV-2 nAbs were reported^12,14,28,33,50,51^. In fact, a study reported that *IGHV*3-53 was the most enriched gene segment, associated with nearly 300 SARS-CoV-2 S-RBD binders^52^. Moreover, the high-potency antibodies in our study (2B11, 1H8, and 1B6) shared almost identical heavy chains, paring with various light chains. On the other hand, 1E10 and 3A9 were encoded by *IGHV*1-69*04 and *IGHV*3-9*01, respectively, with longer HCDR3 lengths (19 amino acids)^53^. Although the correlation between HCDR3 lengths and neutralizing activities of SARS-CoV-2 antibodies remains unknown, these results suggest that the sequence features of highly potent nAbs can be clustered and that the heavy chain probably plays an important role in the blocking of the RBD–ACE2 interactions.

Curiously, although 1E10 antibodies could not block the binding between RBD and ACE2, they showed relative potent and broad-spectrum (pseudo)virus-neutralizing ability. One possible explanation for this phenotype is associated with its encoding gene, *IGHV*1-69, a germline gene commonly associated with antiviral broad neutralizing antibodies (bnAbs)^54^ against viruses such as influenza virus^15,55^, HIV^56^, SARS-CoV^57^, and MERS-CoV^58,59^. These bnAbs target the virus-conserved stalk region containing the glycan pocket; interestingly, studies have found that the hydrophobic tip (positions 53 and 54) in HCDR2 of *IGHV*1-69 is slightly buried inside the pocket and that the long HCDR3 loop enables bnAbs to access the protein surface around the base of the glycan pocket^55^. Another possible explanation is based on the interactions between bnAbs and glycans on the surface of viruses. The SARS-CoV-2 S protein is extensively glycosylated and, therefore, shielded from antibody recognition^1,60^. Of note, the role of glycans is not limited to shielding; they are also important for the transition between the RBD “up” and “down” states, a dynamic process involving the cleavage at the Furin and S2′ sites and conformational changes of multiple domains and the attached glycans^1^. For instance, many glycan-dependent HIV nAbs have been discovered targeting both envelope proteins and glycans. In fact, recently, *IGHV*1-69-encoded monoclonal antibodies were shown to potently neutralize HIV-1 via recognition of the glycan supersite^56^. In line with this, *IGHV*1-69-derived nAbs against the SARS-CoV-2 S protein, such as 1E10 may also neutralize the viruses in vivo via glycan-binding near the RBD–ACE2-binding site, partially hampering the free movement of the RBD or providing steric hindrance. Further crystal structure of 1E10 and RBD complexes will help to understand the exact mechanism.

It is noteworthy that a few nAbs derived from convalescent patients are remarkably similar to the germline segments, without extensive somatic hypermutations. Previously, SARS-CoV-2 S1(RBD)-specific bnAbs also showed low levels of somatic hypermutations^61^. This may be explained by the fact that most of the convalescent patients were in the first wave of the adaptive immune response; naïve B cells contacted with viral antigens for the first time and generated a huge amount of immunoglobulins derived directly from *IGHV* germline genes with few mutations. The usage of pooled PBMCs to generate phage-display libraries limited the analysis of antibody evolution in convalescent patients. However, the lineage, somatic hypermutations, and structural features of nAbs reported in this study may help to set the criteria for the in vitro prediction and screening of SARS-CoV-2 nAbs with high potency, especially in combination with cutting-edge cell sequencing technologies^12^.

NAbs against the SARS-CoV-2 S1 protein have shown promising results as either prophylactic agents against SARS-CoV-2 infection or early-stage therapeutic agents in the context of COVID-19 patients^46,62^. Hypothetically, they can either be used alone or as a complement to vaccines, especially in populations that do not mount strong immune responses to vaccination (e.g., infants, elderly, and immunocompromised individuals). In fact, our in vivo results showed that the early administration of 2B11 resulted in milder pathological lung damage, lighter degree of body weight loss, and lower viral load (vs the administration later after infection, though not achieved a statistical significance), suggesting that the timing of mAbs therapy initiation will affect the treatment efficacy^63^.

In conclusion, using the convalescent patients-derived phage-display libraries, we have obtained a potent nAb 2B11, which exhibited effectively neutralizing ability against WT SARS-CoV-2 and its variants B.1.1.7, and a broad-spectrum nAb 1E10, which could neutralize WT SARS-CoV-2, the variants B.1.1.7, B.1.351 and P.1. Our results support the further development of 2B11, alone or in combination with other distinct epitope-targeted antibodies (such as 1E10), for short-term prevention and early treatment of SARS-CoV-2 infections.

## Materials and methods

### Viruses, cells, and recombinant proteins

A patient-derived SARS-CoV-2 strain (2019-nCoV-WIV04) was used in the neutralization assays of authentic viruses and animal experiments. Original WT pseudoviruses or pseudoviruses containing the full package of P.1, B.1.1.7, or B.1.351 variants were obtained from the Beijing Tiantan Pharmacy Biological Research & Development Company and used in the neutralization assay of pseudoviruses. All experiments with authentic SARS-CoV-2 viruses were carried out in the Biosafety Level 3 facility of the Wuhan Institute of Virology, Chinese Academy of Sciences (CAS).

ACE2-293T cells were generated via the overexpression of the human ACE2 receptor in HEK293T cells and were used in the neutralization assays of authentic viruses. Huh7 (Human hepatoma) cells were used in the neutralization assay of pseudoviruses. Expi293F cells (Thermo Fischer Scientific) were cultured in Expi293™ Expression Medium (Thermo Fischer Scientific) to express antibodies. Additionally, Expi293F, ACE2-293T cells, and Vero E6 cells were cultured in Dulbecco’s modified Eagle’s medium (DMEM, Gibco, Thermo Fischer Scientific) supplemented with 10% fetal bovine serum (FBS), penicillin (100 U/mL), and streptomycin (100 U/mL).

Recombinant SARS-CoV-2 proteins including the S1-His protein (Sino; 40591-V08H), RBD-mFc protein (Sino, 40592-V05H), S1-hFc protein (Sino, 40591-V02H), the mutant RBD-His proteins including N354D (Sino, 40592-V08H2), A435S (Sino, 40592-V08H4), V483A (Sino, 40592-V08H5), F342L (Sino, 40592-V08H6), K17N (Sino, 40592-V08H59), K458R (Sino, 40592-V08H7), G476S (Sino, 40592-V08H8), W436R (Sino, 40592-V08H9), N501Y (Sino, 40592-V08H82), E484K (Sino, 40592-V08H84), E484K/K417N/N501Y (AtaGenix, ATMP02511COV), E484K/K417T/N501Y (AtaGenix, ATMP02513COV) or WT RBD-His (Sino, 40592-V08H), and the recombinant mutant S1-His protein D614G (Cusabio, MP3324GMY) or control S1-His (Sino, 40591-V08H) were used in the context of phage-display library panning or ELISA, together with the human ACE2-His protein (Sino, 10108-H08H), ACE2-Fc (Sino, 10108-H02H). The recombinant SARS-CoV-2 S1 protein (Kactus Biosystems, COV-VM4S1) was used in SPR. The Phagemid vector PFL249, containing hexahistidine and c-myc tag encoding sequences linked by a 21-amino acid-encoding peptide (G4S)3GGGGAS, was used to construct the phage-display libraries.

### Blood samples and processing

Eight COVID-19 convalescent patients were recruited, and blood samples (10 mL per individual) were collected after written informed consent was obtained. PBMCs were isolated using a Ficoll Paque Plus (GE Healthcare, Sweden) density gradient media and stored at −80 °C in liquid nitrogen until use; serum was also collected to determine anti-RBD antibody titers.

### Determination of anti-RBD antibody titers via ELISA

An ELISA was used to determine the RBD-specific antibody titers in the serum of convalescent patients. In brief, 50 ng/well of recombinant RBD was used to coated 96-well plates in carbonate–bicarbonate buffer overnight at 4 °C. Plates were then washed with PBST (PBS with 0.5% (v/v) Tween 20). After 1 h of blocking with casein buffer, serially diluted serum samples were added to each well and incubated for 1 h at 37 °C. Plates were then washed with PBST and incubated with 1:10,000 diluted horseradish peroxidase (HRP)-goat anti-human IgG antibodies (Bethyl Laboratories, A80-304P) for 1 h at 37 °C. After washing with PBST, 100 μL/well tetramethylbenzidine (TMB) substrate was added for detection. The reaction was stopped via the addition of 100 μL/well of 2 M HCl. The absorbance was measured at 450 nm using an ELISA reader, and the data were analyzed using the GraphPad Prism software v. 8.0.1. Serum of healthy donors was used as control, and positive was defined as higher the cut-off values (2.1 × the absorbance at 450 nm of control).

### Construction of the phage-display libraries

Two independent phage-display libraries (scFvHκ and scFvH_λ_) were constructed. In brief, total RNA was extracted from PBMCs using the TRIzol reagent (Invitrogen, USA), and then cDNAs were synthesized using the SuperScript III first-Strand Synthesis System Kit (Invitrogen). The VH, VL_κ_, and VL_λ_ regions of antibodies were separately amplified from the obtained cDNAs using the PrimerSTAR DNA polymerase (Takara, Kyoto, Japan) and then cloned into the Phagemid vector PFL249. VH/VL_κ_ or VH/VL_λ_-containing Phagemid vectors were then transformed by electroporation into *E. coli* (Lucigen, USA) and subjected to culture on YT-GC agar plates overnight at 37 °C. Individual libraries were stored as glycerol stocks (in aliquots) in liquid nitrogen until use. In addition, for the determination of the insertion rate, 96 colonies were randomly picked, and colony-PCR with the M13-48 (5′-GAGCGGATAACAATTTCACACAGG-3′) and pFL249-R (5′-AGCCCCCTTATTAGCGTTTGC-3′) primers was performed (target fragment of about 1 kb). Finally, yet importantly, for the determination of phage titers, phage libraries were amplified via helper phage infection, purified using PEG precipitation, and subjected to gradient dilution.

### Panning and screening of the phage-display libraries

Three rounds of panning were performed using the target antigens, including recombinant S1-hFc and RBD-mFc. Briefly, phage-display libraries were incubated in 10 μg/mL target antigen-coated immunotubes for 1 h at room temperature (RT). After washing extensively with PBST, the bound phages were eluted with 0.1 M Gly-HCl buffer (pH 2.2). The eluted phages were used to infect *E. coli* for subsequent amplification and subjected to further selection rounds. In the second and third rounds of panning, the input phages or output phages were allowed to incubate once or twice with human or mouse IgG mixtures-adsorbed immunotube for 1 h at RT for Fc tag antibody depletion.

After panning, the output clones were picked, induced, and screened via monoclonal soluble scFv ELISA with the S1 protein and human IgG mixture, or the RBD protein and mouse IgG mixture, respectively. In brief, 96-well plates were coated with 2 μg/mL of S1 protein (100 μL per well), or 1 μg/mL of RBD protein (100 μL per well), and 1.5 μg/mL (100 μL per well) of human IgG mixture and mouse IgG mixture diluted in NaHCO_3_ (pH 9.2) overnight at 4 °C. The coated plates were blocked with 2% milk for 1 h at RT and washed with PBST. Then, plates were incubated for 1 h with soluble scFv induced in the supernatant from individual clones. After incubation, plates were washed with PBST and incubated with HRP-conjugated anti-c-Myc antibody for 1 h at RT. Before reading, the plates were washed with PBST and developed with TMB peroxidase substrate solution. The reactions were stopped with 2 M HCl, and plates were read at 450 nm.

### Sequencing and genetic analyses of phage antibodies

The positive hits were obtained and sent for unique clone identification via sequencing. The V gene families were assigned using the internal analysis website, which was developed based on the Ig-Blast server at NCBI (http://www.ncbi.nlm.nih.gov). Furthermore, the unique clones were subjected to the second round of screening based on the binding confirmation by ELISA.

### Competition FACS assay using ACE2-expressing cells

HEK293T-hACE2 cells, engineered in-house, were seeded at 1 × 10^5^ cells/well in U-bottom 96-well plates, cultured for 24 h, and then centrifuged to discard the supernatant. In all, 0.2 μg/mL RBD-mFc in PBS was mixed with diluted culture supernatant of positive clone, and then added to the plates containing HEK293T-hACE2 cells. After incubation at 4 °C for 1 h and washing, PE-labeled goat anti-mouse IgG Fc antibodies were added. After washing and subsequent re-suspension, the cells were acquired by flow cytometry, and the mean fluorescence intensity (MFI) was analyzed using the FlowJo software.

### Antibody cloning and expression

The variable region of each antibody was synthesized by PCR from scFv plasmids and cloned into modified pcDNA3.4 expression vectors together with the constant region of human IgG1. Expi293F cells were transiently transfected with recombinant pcDNA3.4 plasmids for antibody expression and cultured at 37 °C, in an 8% CO_2_ atmosphere. The supernatants were then collected 5 days post transfection for SDS-PAGE analysis and purification using protein A affinity chromatography (MabSelect Sure resin). The concentration and purity of the antibodies were determined using the NanoDrop at 280 nm and HPLC-SEC, respectively.

### Binding ELISA

ELISA was performed to evaluate whether the antibodies could bind to SARS-CoV-2 RBD-mFc or S1-His proteins. In brief, the S1 or RBD proteins were used to coat 96-well ELISA plates (Nunc MaxiSorp, Thermo Fisher Scientific) at 1.0 μg/mL in carbonate–bicarbonate buffer overnight at 4 °C. After washing with PBST and blocking with casein buffer, serially diluted antibodies in casein buffer were added to each well and incubated for 1 h at RT. Plates were then washed with PBST and incubated with a 1:10,000 dilution of HRP-goat anti-human IgG antibody for 1 h at RT. After washing, 100 μL/well TMB substrate was added for detection. The reaction was stopped with 100 μL/well of 2 M HCl and the absorbance was measured at 450 and 540 nm using an ELISA reader.

### Competition ELISA

The blocking ability of the antibodies in the context of hACE2 was evaluated using ELISA. Briefly, SARS-CoV-2 S1-hFc or RBD-mFc were used to coat 96-well ELISA plates at 1.0 μg/mL in carbonate–bicarbonate buffer overnight at 4 °C. After washing with PBST and blocking with casein buffer, serial dilutions of antibodies (3-fold dilutions, from 20.0 μg/mL to 0.001 μg/mL) and the ACE2-His (0.5 μg/mL or 0.02 μg/mL) protein in casein buffer were co-incubated on the plates for 1 h at RT. Plates were then washed with PBST and incubated with a 1:3000 dilution of biotin-labeled anti-His antibody (Genscript, A00613) for 1 h at RT. After washing, 1:20,000 streptavidin–HRP (Invitrogen, SNN1004) was added to the plates and incubated for 1 h at RT. After washing with PBST, 100 μL/well TMB substrate was added. The reaction was stopped with 100 μL/well of 2 M HCl and the absorbance was measured at 450 nm and 540 nm using an ELISA reader.

A similar assay was used to detect the blocking ability of the 2B11 antibodies against different RBD or S1 mutants. In brief, 2 μg/mL ACE2-Fc was used to coat the 96-well ELISA plates. Recombinant mutant RBD-His proteins including N354D, A435S, V483A, F342L, K458R, G476S, W436R, N501Y, E484K, E484K/K417N/N501Y, E484K/K417T/N501Y, or WT RBD-His (740 ng/mL, 50 μL/well) were used as the competitors, together with twofold serially diluted mAb 2B11 starting from the initial concentration of 15,000 ng/mL (50 μL/well). Recombinant mutant S1-His protein D614G or control S1-His were also used as competitors (10 μg/mL, 50 μL/well), together with threefold serially diluted mAb 2B11 (50 μL/well) starting from the initial concentration of 25,000 ng/mL. The protocol used is the same as above.

### SPR

Antibodies were captured on an anti-human IgG Fc antibody-immobilized CM5 sensor chip (29-1496-03; Chicago, IL, USA). The recombinant SARS-CoV-2 S1 protein at different concentrations was then injected over the sensor chip at a flow rate of 30 μL/min for an association phase, followed by a dissociation phase. The chip was regenerated using 10 mM glycine (pH 1.5) after each binding cycle. The measurements of the blank surface and buffer channel were subtracted from the test measurements. The experimental data were fitted using a 1:1 binding model. Molecular weight of 77.9 kDa was used to calculate the molar concentration of the S1 protein.

### Pseudovirus-neutralization assay

Pseudovirus-neutralization assays were performed as previously described^64^. In brief, serially diluted mAbs were incubated with the SARS-CoV-2 pseudoviruses for 1 h at 37 °C, together with the control groups without antibodies and/or viruses. The mixtures were subsequently incubated with Huh7 cells for 24 h under a 5% CO_2_ atmosphere at RT. In total, 100 μL of the culture supernatant were then replaced with luciferase substrate, and 2 min of incubation at RT 150 μL of the lysate was transferred into a new 96-well plate to detect luciferase activity using the Nano-Glo Luciferase Assay System (Promega). Relative luminescence units were normalized to those derived from cells infected with SARS-CoV-2 pseudoviruses in the absence of monoclonal antibodies. The IC_50_ was determined via a four-parameter logistic model using the GraphPad Prism v. 8.0.1.

### Authentic SARS-CoV-2 neutralization assay

The plaque reduction neutralization test (PRNT) was carried out in the Biosafety Level 3 facility of the Wuhan Institute of Virology, CAS, using a clinical isolate of SARS-CoV-2 (2019-nCoV-WIV04). Briefly, 0.3 mL of fourfold serial dilutions of mAbs were added to the same volume of ~600 pfu/mL of SARS-CoV-2 and incubated for 1 h at 37 °C. The initial concentration of the mAbs varied from 4000 ng/mL for 1F2, 1B6, 2B11, 1H8, and 1D7, to 5000 ng/mL for 1E10 and 1D10, and to 50,000 ng/mL for 2A2, 1B10, and 3A9. The 0.5 mL mixtures were then added to a monolayer of Vero cells in six-well plates and incubated for 1 h at 37 °C. The mixture was removed, and 1.0 mL of 0.9 % (w/v) LMP agarose (Promega) in 2× DMEM supplemented with 4% (v/v) FBS was added onto the infected cells. After further incubation at 37 °C in a 5% CO_2_ atmosphere for 2 days, the wells were stained with 0.05% (w/v) crystal violet dissolved in 8% (v/v) formaldehyde to visualize the plaques. The IC_50_ values were determined using the Reed-Mench method.

### Epitope binning via bio-layer interferometry

To assess epitope specificity, mAbs were binned using an Octet Red 96 system. Briefly, 1 µg/mL of the RBD-His recombinant protein was captured using a HIS1K sensor (ForteBio, 18-5120) for 1 min, and then, a saturating concentration of mAb (200 nM) was added for 3 min. Competing concentrations of mAbs (200 nM) were then added for 3 min to measure the binding in the presence of saturating antibodies. All incubation steps were performed in pH 7.4, 1× PBS containing 0.1% BSA, and 0.02% Tween.

### Crystallization

To obtain 2B11-Fab, purified 2B11 (2 mg/mL) was incubated with papain (0.1 mg/mL) in PBS with 5.5 mM cysteine and 2 mM EDTA for 1.5 h at 37 °C. The reaction was stopped with 25 mM iodoacetamide, and 2B11-Fab was purified via protein A-based chromatography.

The 2B11-Fab fragment and SARS-CoV-2 RBD protein (Sino, 40592-VNAH) were mixed at a molar ratio of 1.2:1, incubated on ice overnight, and then purified using Superdex 200 (GE Healthcare) and the SEC buffer (20 mM Tris-HCl, pH 8.0, 150 mM NaCl). The eluate was concentrated to 10 mg/mL for crystal screening by the sitting-drop method at 4 °C. The following kits were used for screening, including the PEG/Ion, PEG/Ion2, Index, SaltRX, Crystal Screen, and Crystal Screen2 (Hampton Research).

After 3 weeks, flat-like crystals appeared in the mother liquid containing 0.2 M ammonium sulfate, 12% w/v PEG 8000, and 0.1 M Tris-HCl, pH 8.5. The crystal growth conditions were further optimized using the sitting-drop method with a volume ratio of 1:1. Crystals were cryo-protected in the mother liquid containing 20% v/v ethylene glycol and cooled in a dry nitrogen stream at 100 K for X-ray diffraction.

### X-ray data collection, processing, and structure determination

The diffraction data were collected (BL17U1) in the Shanghai Synchrotron Radiation Facility (wavelength, 0.97919 Å) at 100 K. The diffraction data were auto-processed using the aquarium pipeline. The structure was solved by molecular replacement with the previously reported SARS-CoV-2 RBD and Fab structures (PDB ID: 7C01). The initial model was completed using Coot^65^ and further refined with phenix.refine using Phenix^66^. All structural figures were generated using the Pymol software.

### Animal experiments

The effectiveness of 2B11 antibodies against SARS-CoV-2 infection in vivo was investigated in AdV5-hACE2-transduced, 8-week-old, SPF, male C57BL/6 J IFNAR^–/–^ mice. Briefly, the mice were intranasally transduced with 4 × 10^8^ 50% cell culture infectious dose (TCID_50_) of AdV5-hACE2 to induce the expression of hACE2 in the lungs. Five days post transduction, mice were challenged with 1 × 10^6^ TCID_50_ of SARS-CoV-2 via the intranasal route and monitored over a 6-day time course. Two independent experiments were performed to determine the neutralization ability of different doses of 2B11 antibodies against SARS-CoV-2 in vivo; transgenic mice were divided into different groups: prophylactic, therapeutic, and placebo groups. In the low-dose experiment, 2B11 (25 mg/kg) antibodies were injected intraperitoneally 24 h before or 12 h after SARS-CoV-2 challenge in mice in the prophylactic (*n* = 6) or therapeutic (*n* = 6) groups, respectively, while PBS (200 μL) was injected intraperitoneally 12 h after SARS-CoV-2 challenge in animals in the placebo group (*n* = 6). In the high-dose experiment, 75 mg/kg 2B11 was injected intraperitoneally, 24 h before (*n* = 7), or either 2 h (*n* = 7) or 12 h (*n* = 7) after the SARS-CoV-2 challenge; the placebo group (*n* = 7) received PBS intraperitoneally 12 h after SARS-CoV-2 challenge. The bodyweight of the animals was recorded daily for 6 consecutive days. The mice were euthanized at 6 days post challenge, and their lungs were collected for histology and viral load analyses.

Viral load detection was performed by quantitative real-time polymerase chain reaction (qRT-PCR) with RNA extracted from lung homogenate supernatants. Briefly, lung homogenates were prepared via homogenization of the collected lungs using an electric homogenizer; and the total RNA from the homogenates was extracted using the TRIzol. One microgram of RNA was used for reverse transcription through the PrimeScript RT reagent Kit with gDNA Eraser (Takara, RR047Q); then the qRT-PCR was performed (two replicates per animal) using the TB Green Premix (Takara, RR820A) on a StepOne Plus Real-time PCR instrument (Applied Biosystems), according to the manufacturer’s instructions. Two microliters of standard or cDNA template were utilized for qPCR using the RBD primers (RBD-qF: 5′-CAATGGTTTAACAGGCACAGG-3′; RBD-qR: 5′-CTCAAGTGTCTGTGGATCACG-3′). The amplification was performed as follows: 95 °C for 15 s followed by 40 cycles consisting of 95 °C for 15 s, 54 °C for 15 s, and 72 °C for 30 s.

For histology analysis, the collected lung tissues were fixed in 10% neutral formalin buffer for more than 3 days and sent for paraffin embedding and sectioning. The sections were stained with hematoxylin and eosin and observed under a light microscope. According to the pathological characteristics of the lungs, the damage level of the organs and tissues was determined and divided into three disease groups: severe, moderate, and mild.

### Statistical analysis

The experimental data were analyzed with GraphPad Prism 8.0 software (GraphPad Software Inc.). In animal experiments, the comparison of body weight changes and viral load changes were performed using two-tailed Student’s *t-*test, and *P* values < 0.05 were considered statistically significant. Data were expressed as the mean ± SD.

## Supplementary information

Supplementary Information
