# Supplementary material for: Screening of potent neutralizing antibodies against SARS-CoV-2 using convalescent patients-derived phage-display libraries
Source: Cell Discov. 2021 Jul 27;7:57. doi: 10.1038/s41421-021-00295-w (PMC8315086; doi:10.1038/s41421-021-00295-w)

Supplementary

Fig. S1

Binding activity of 2B11against mutant proteins. **(a-c)** Binding activity of 2B11 against mutant proteins. **(d-e)** Binding activity of RBD against hACE2 proteins.

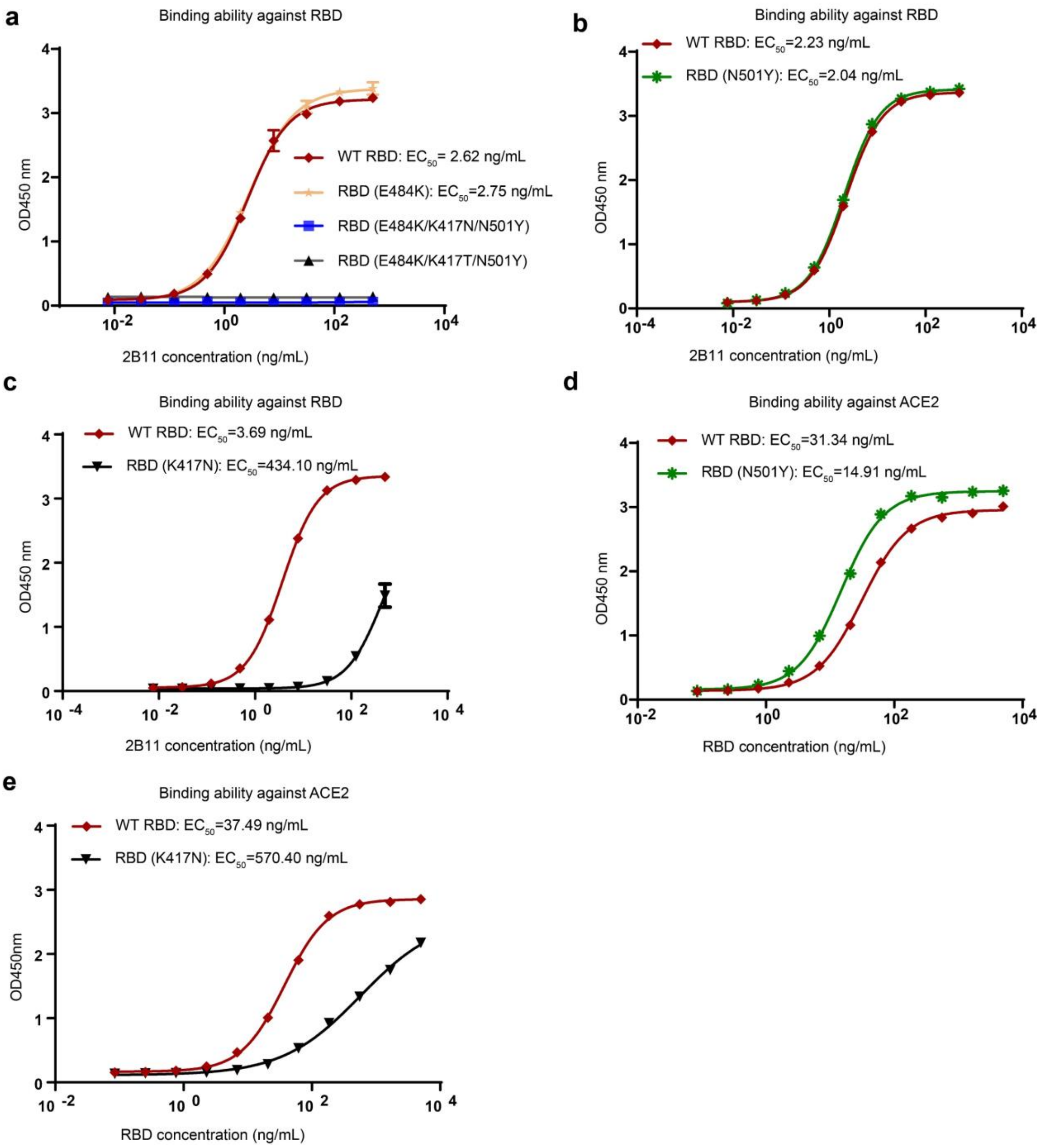

Supplement: Supplementary file 1 — Supplementary Information [file 41421_2021_295_MOESM1_ESM.pdf]
